# Complete home smoking ban survey analysis: an opportunity to improve health equity among sexual minority adults in California, USA

**DOI:** 10.1186/s12889-022-12891-w

**Published:** 2022-03-18

**Authors:** Marie C. Boman-Davis, Veronica L. Irvin, Erika Westling

**Affiliations:** 1 Oregon Research Institute, 1776 Millrace Dr., Eugene, OR 97403 USA; 2 Oregon State University, College of Public Health and Human Sciences, School of Social and Behavioral Health Sciences, Corvallis, OR USA

**Keywords:** Home smoking bans, Sexual minority

## Abstract

**Background:**

Increasing the proportion of adults living in smoke-free homes is a US Healthy People 2020 objective. Complete home smoking bans are associated with higher odds of smoking cessation attempts and cessation duration. Sexual minority adults have disproportionality higher rates of smoking. This study investigates correlates of having a complete home smoking ban among sexual minority adults in California.

**Methods:**

Secondary data analyses of the California Behavioral Risk Factor Surveillance System (CA BRFSS), 2014–2016. The CA BRFSS telephone survey of adults was conducted in English and Spanish and used random digit dial for landline and cell numbers. Weighted descriptives were stratified by sexual orientation and biological sex. Weighted bivariate and multivariable logistic regression analyses included only sexual minorities (i.e., lesbian, gay, bisexual) and were analyzed as a group and separately by biological sex to account for intragroup variances. The final weighted total of sexual minority adults (*N* = 359,236) included sexual minority adult females (*N* = 163,490) and sexual minority adult males (*N* = 195,746).

**Results:**

Sexual minority adults in California had a lower prevalence of complete home smoking bans (Female 76.2%; Male 75.7%), higher prevalence of current cigarette smoking (Female 23.3%; Male 17.4%) and of e-cigarette use (Female 5.8%; Male 6.4%) than their straight counterparts. Sexual minorities that smoke everyday (Female Adjusted Odds Ratio (AOR) 0.26, 95% Confidence Interval (CI) 0.11–0.63; Male AOR 0.24, 95% CI 0.01–0.56) or some days (Female AOR 0.28, 95% CI 0.09–0.90) had lower adjusted odds of having a complete home smoking ban compared to those who never smoked.

**Conclusions:**

Smoking everyday was the only consistent predictor of not having a complete home smoking ban among sexual minority adults. Focused efforts to increase prevalence of complete home smoking bans should address smoking status to improve health equity among sexual minority adults.

## Background

Healthy People 2020 tobacco use objectives included increasing the proportion of adults living in smoke-free homes to 87% and decreasing adult smoking prevalence [1]. This strategy was designed to improve population health and wellbeing, including the health of sexual minorities. Adoption of voluntary home smoking bans have been influenced by a variety of factors, including public policy smoking restrictions [2, 3]. Although banning the use of electronic (e-) cigarettes was not explicitly included in the national smoke-free homes objective, there was a consensus among national and international public health organizations (e.g., the World Health Organization) that smoking bans should also include use of e-cigarettes and vaping [4, 5]. Previous research has focused on population prevalence of bans and the relationship of bans with tobacco cessation; however, little is known about how individual level characteristics, such as sexual minority status, smoking status, or use of e-cigarettes, may interact with home smoking bans. Complete home smoking bans reduce exposure to secondhand and thirdhand smoke for household members, pets, and visitors and increase smoking cessation and reduction in smoking for those who smoke [6, 7]. As sexual minorities have disproportionately high rates of smoking and vaping, it is important to examine rates of smoking, vaping, and smoking bans within this population, to inform policies to increase health equities.

Sexual minorities reported higher use of all forms of tobacco compared with their straight counterparts [8–11]. In 2017 National Health Interview Survey, the prevalence of any tobacco use was higher among lesbian, gay, or bisexual adults (27.3%) than among straight adults (19.0%) [9]. Lesbian and gay adults had statistically significantly higher odds of reporting current smoking and everyday smoking than straight adults [12]. In 2014–2017 Behavioral Risk Factor Surveillance System, lesbian/gay and bisexual adults had significantly higher odds of reporting everyday smoking and current smoking (everyday or some days) compared to straight adults [12]. The prevalence of current e-cigarette use was most prevalent among US sexual minority adults [8, 10, 13]. Data from the 2016 Behavioral Risk Factor Surveillance System reported among data from 198,057 adults in 26 states that lesbian, gay, and bisexual adults were significantly more likely to ever and currently smoke cigarettes and e-cigarettes compared to straight adults [8]. Data from the US Behavioral Risk Factor Surveillance System in 2016, 2017, and 2018 (*n* = 1,348,091) reported the prevalence of e-cigarette use among lesbian, gay, bisexual, or transgender (LGBT) adults was 13%, nearly twice that of straight adults (5%) [13]. In the Population Assessment of Tobacco and Health in 2013–2014, lesbian/gay and bisexual women had higher odds of regular use of cigarettes and e-cigarettes [10].

Examining how smoking bans interact with smoking status of sexual minority adults can inform how health policies may be leveraged to reduce persistent disparities in tobacco use among LGBT adults compared to straight adults [6]. In 2015, the national prevalence of smoke-free homes reached 86.5%, and California exceeded the 87% goal, with 91.7% of adults living in smoke-free homes (as cited in [14]). The prevalence of complete home smoking bans among sexual minorities nationally and in California remains unknown, as do the characteristics and correlates of sexual minorities with complete home smoking bans. Thus, the objective of this study is to investigate the presence of a complete home smoking ban among sexual minority adults in California, after adjusting for current smoking status, e-cigarette use, socio-demographics, and other characteristics. Male and female respondents were analyzed separately to account for intragroup variances.

## Methods

### Study design

This secondary data analysis study combined cross-sectional data of the California Behavioral Risk Factor Surveillance System (CA BRFSS), collected in 2014, 2015 and 2016 and included comparable measurement of household smoking rules. The CA BRFSS telephone survey of adults was conducted in English and Spanish and used random digit dialing for landline and cell numbers of non-institutional adult populations with geographic stratified sampling and continuous data collection [15]. Respondents with recorded answers for all study variables were included in the present analyses.

### Stratification variables

Present study analyses were stratified by sex assigned at birth and sexual orientation, using the CA BRFSS dataset variables “Sex,” and “Self-Reported Sexual Orientation” [15]. In survey years 2014 and 2015, sex was asked as “Are you Male or Female?” In survey year 2016, the question was revised and asked as “Sex Assigned to You at Birth, On Birth Certificate” [15]. Male and female respondents were also analyzed separately to account for intragroup variances. In survey years 2014 and 2015, sexual orientation collected through one question that asked, “Do you consider yourself to be...” with the following answer options ‘Heterosexual, That is Straight’, ‘Homosexual, That is Gay or Lesbian’, ‘Bisexual’, ‘Other’, ‘Don’t Know’ and ‘Refuse to Answer’. In survey year 2016 the answer options included ‘Straight’, ‘Lesbian or Gay’, ‘Bisexual’, ‘Other’, ‘Don’t Know’ and ‘Refuse to Answer’ [15] Sexual minority in this study included participants who identified as lesbian, gay, or bisexual (LGB).

### Explanatory variable

The original categorical variable was “Cigarette Smoking Status,” with values ‘Currently Smokes (Everyday)’, ‘Currently Smokes (Some days)’, ‘Formerly Smoked’, and ‘Never Smoked’ (reference group) [15]. The categorical variable “Cigarette Smoking Status,” was retained; however, values were renamed ‘Smokes Cigarettes Everyday’, ‘Smokes Cigarettes Some days’, ‘Formerly Smoked Cigarettes’, and ‘Never Smoked Cigarettes’ (reference group) to clearly identify cigarette smoking and not e-cigarette smoking.

### Outcome variable

The outcome variable selected from the CA BRFSS datasets was “Smoking Rules in Household” with values ‘Completely Prohibited’; ‘Generally Prohibited’; ‘Allowed in Some Rooms Only’; and ‘No Restrictions on Smoking’ [15]. The categorical variable was dichotomized to measure a complete home smoking ban previously defined as no smoking allowed in the home ever. The final variable was labeled “Complete Ban” and the values were ‘Yes’ and ‘No’ (reference group).

### Covariates

Covariates included the following: a) e-cigarette use; b) marital status; c) age group; d) educational attainment; e) status of children (< 18) in the home; f) income, g) race/ethnicity; and h) survey year. The continuous measure of e-cigarette use in the past 30 days was dichotomized and the final variable was “E-cigarette Use (Past 30 Days)” with values ‘Yes’ and ‘No’ (reference). The categorical variable “Marital Status” included ‘Never Married’, ‘Unmarried Couple’, ‘Divorced/Widowed/Separated’ and ‘Married’ (reference). The categorical variable “Age Group” included ‘18–24’, ‘25–44’, ‘45–64’ and ‘65+’ (reference). The categorical variable “Educational Attainment” included ‘Fewer than 12 Years of Education (No Diploma)’, ‘High School Graduate (Diploma or GED)’, ‘Some College or Technical School’, and ‘College Graduate’ (reference). The dichotomous variable “Status of Children (<18) in the Home” included ‘No Children in Household’ and ‘Yes - At least 1 Child in Household’ (reference). The categorical variable for “Income” included ‘< $15,000’, ‘$15,000-$24,999’, ‘$25,000–$34,999’, ‘$35,000–$49,999’, and ‘$50,000+’ (reference). The categorical “Race/Ethnicity” variable included ‘Black Only, Non-Hispanic’ ‘Other Race Only, Non-Hispanic’, ‘Multiracial, Non-Hispanic’, ‘Hispanic’ and ‘White Only, Non-Hispanic’ (reference). The ordinal variable “Survey Year” included ‘2014’ (reference), ‘2015’ and ‘2016’.

### Analysis

Weighted analyses included descriptive, bivariate (i.e., Pearson chi-square, alpha = 0.05), and a multivariable logistic regression. CA BRFSS survey weights reflected estimates from the State of California’s Department of Finance [15]. Data management and analyses were conducted with Base SAS version 9.4 (Cary, NC, USA). Waivers were granted by the Institutional Review Boards at both the Oregon Research Institute and Oregon State University, as the dataset was de-identified.

## Results

**Fig. 1:**
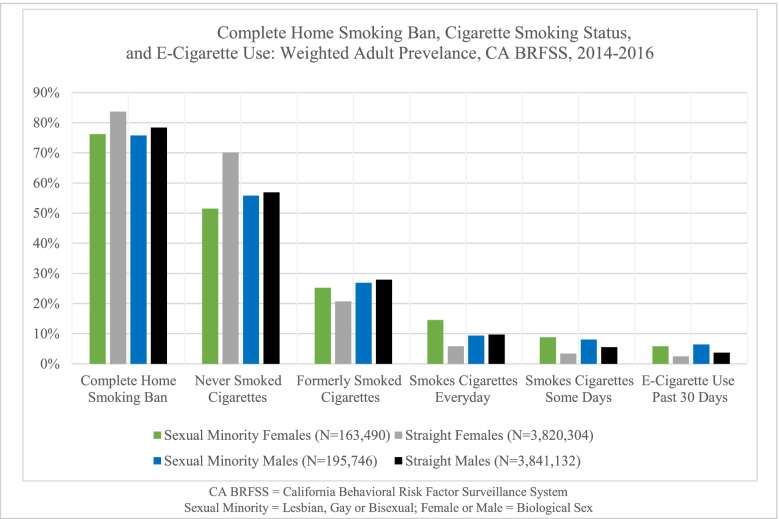
Complete home smoking ban, Cigarette smoking status, and E-Cigarette use: weighted adult prevelance, CA BRFSS, 2014-2016

The final weighted populations in California were sexual minority females (*N* = 163,490), sexual minority males (*N* = 195,746), straight females (*N* = 3,820,304), and straight males (*N* = 3,841,132). Sexual minorities had a lower prevalence of complete home smoking bans (Female = 76.2%; Male = 75.7%) compared to straight adults (Female = 83.7%; Male = 78.4%). Female sexual minorities had the highest prevalence of smoking cigarettes everyday (14.5%) and some days (8.8%; Fig. 1). Male sexual minorities had the highest prevalence of past 30-day e-cigarette use (6.4%), followed by female sexual minorities (5.8%), straight males (3.7%) and straight females (2.5%).

Sexual minorities that smoke cigarettes everyday had 75% lower adjusted odds ratios (AOR) and those that smoke cigarettes some days had more than 64% lower AORs of having a home with a complete smoking ban compared to those that never smoked (Table 1). Similarly, female sexual minorities that smoke everyday had 74% lower AORs and those that smoke cigarettes some days had 72% lower AORs of having a home with a complete smoking ban compared to those who never smoked (Table 1). Male sexual minorities that smoke everyday had 76% lower AORs of having a home with a complete smoking ban compared to those who never smoked (Table 1).

**Table 1:** Complete home smoking ban and smoking among sexual minority adults: weighted regressions, CA BRFSS 2014–2016

			**Sexual Minority◊ Adults** **Complete Home Smoking Ban**					
	**Weighted Population**		**Yes**		**No**		**AOR**^a^	**95% CI**^b^
	N	(%)	n	(%)	n	(%)		
	359,236	(100)	272,736	(75.9)	86,500	(24.1)		
**Cigarette Smoking Status**								
Smokes Cigarettes Everyday^1^	42,117	(11.7)	22,461	(53.3)	19,656	(46.7)	**0.26**	**0.15–0.46**
Smokes Cigarettes Some Days^1^	30,041	(8.4)	18,369	(61.1)	11,645	(38.8)	**0.36**	**0.17–0.76**
**Income**								
<$15,000^2^	71,507	(19.9)	48,606	(68.0)	22,901	(32.0)	**0.42**	**0.23–0.76**
$25,000–$34,999^2^	28,304	(7.9)	18,135	(64.1)	10,169	(35.9)	**0.30**	**0.14–0.66**
**Race/Ethnicity**								
Black, Non-Hispanic^3^	12,845	(3.6)	7194	(56.0)	5651	(44.0)	**0.34**	**0.14–0.84**
**Survey Year**								
2016^4^	97,175	(27.1)	86,401	(88.9)	10,773	(11.1)	**4.20**	**2.26–7.76**
			**Sexual Minority◊ Adult Females^** **Complete Home Smoking Ban**					
	**Weighted Population**		**Yes**		**No**		**AOR**^a^	**95% CI**^b^
	N	(%)	n	(%)	n	(%)		
	163,490	(100)	124,525	(76.2)	38,965	(23.8)		
**Cigarette Smoking Status**								
Smokes Cigarettes Everyday^1^	23,742	(14.5)	13,647	(57.5)	10,095	(42.5)	**0.26**	**0.11–0.63**
Smokes Cigarettes Some Days^1^	14,389	(8.8)	8625	(59.9)	5764	(40.1)	**0.28**	**0.09–0.90**
**Income**								
$25,000–$34,999^2^	15,967	(9.8)	2190	(13.7)	7064	(44.2)	**0.21**	**0.07–0.70**
**Race/Ethnicity**								
Black, Non-Hispanic^3^	7270	(4.5)	3683	(50.7)	3587	(49.3)	**0.24**	**0.08–0.75**
**Survey Year**								
2016^4^	40,254	(24.6)	34,622	(86.0)	5632	(14.0)	**2.99**	**1.22–7.34**
			**Sexual Minority◊ Adult Males^** **Complete Home Smoking Ban**					
	**Weighted Population**		**Yes**		**No**		**AOR**^a^	**95% CI**^b^
	N	(%)	n	(%)	n	(%)		
	195,746	(100)	148,211	(75.7)	47,535	(24.3)		
**Cigarette Smoking Status**								
Smokes Cigarettes Everyday^1^	18,375	(9.4)	8813	(48.0)	9561	(52.0)	**0.24**	**0.01–0.56**
**Survey Year**								
2016^4^	56,920	(29.1)	51,799	(91.0)	5141	(9.0)	**5.18**	**2.18–12.29**

^◊ ^Lesbian, Gay or Bisexual; ^**^**^ Biological Sex; ^a^ Adjusted Odds Ratio; ^b^ Confidence Interval

^1^Reference = Never Smoked Cigarettes; ^2^ Reference = $50,000+; ^3^ Reference = White, Non-Hispanic; ^4^ Reference = 2014

Notes: Only statistically significant adjusted odds ratios were included in the table. Variables included in the model with non-significant results included the following: a) E-Cigarette Use (Past 30 Days); b) Sex; c) Marital Status: d) Age Group; e) Educational Attainment; f) Sexual Orientation; and g) Status of Children (<18) in the Home

## Discussion

This is the first paper to investigate home smoking bans and smoking status among sexual minorities. Using CA BRFSS data collected from 2014 to 2016, we extended previous research findings that sexual minorities were less likely to have complete home smoking bans compared to straight adults. Current daily smoking was the strongest predictor of not having a complete home smoking ban among sexual minorities as a group and stratified by sex. Although male sexual minorities had smoking rates comparable to straight adults, female sexual minorities had highest rate of smoking and male sexual minorities had rates of e-cigarette use compared to straight adults. As complete home smoking bans are directly related to health outcomes [6, 7], examining characteristics associated with the presence or absence of bans for sexual minority adults can inform policies designed to increase health equity.

Higher rates of tobacco use among sexual minorities have been linked to poor mental health and well-being, life dissatisfaction, stressors and discrimination/victimization related to sexual orientation [16, 17]. Permissive community norms and nonjudgement towards tobacco use also relate to high rates of use and serve as barriers to cessation attempts [18]. Sexual minority populations often experience a pro-tobacco bar culture that accepts and promotes tobacco use, as well as alcohol use [19]; this also increases environmental smoke and aerosol exposure. Additionally, among same-sex couples, having a partner who reported daily or intermittent smoking had increased odds of current smoking [20].

Our findings have policy implications, especially in conjunction with findings by Wintemberg and colleagues [5] that sexual gender minorities that smoked reported greater intention to quit if they lived in a smoke-free community with a smoke free policy active for at least 2 years compared to a community without a smoke-free policy. Thus, there may be an effect of normative or role-model behavior on smoking.

### Limitations

The present study used cross-sectional data therefore the temporality of smoking status and home smoking bans were indeterminant; however, the adjusted relationships between the variables were investigated. Study findings are limited to California and may not be generalizable to other states. Survey data used in this study only measured LGB therefore it may not be generalizable to the LGBT community. Living in communities with less LGBT-supportive environmental and community norms could increase the stress and thereby substance use and increase the disparity of home bans [16]. An additional study limitation was that the wording changed for several questions in 2016 that may result in some misclassifications. In survey years 2014 and 2015, sex was asked as “Are you Male or Female,” and in 2016 it was changed to “Sex Assigned to You at Birth, On Birth Certificate” [15]. Similarly, in survey years 2014 and 2015, e-cigarette use was asked as “Past 30 Days, # Days Used Electronic Cigarettes” but it was expanded in 2016 to “Past 30 Days, # Days Used E-Cig or Vape Pen, Tank, or Mod” [15]. Thus, prevalence of e-cigarette use may have been underreported prior to 2016.

## Conclusion

Focused efforts to increase prevalence of complete home smoking bans should address smoking status to improve health equity among sexual minority adults. First, expanding and strengthening community resources and social norms for LGB is recommended to improve the well-being of the community and possibly reduce substance use disparities [21]. Second, comprehensive approaches should include implementation of community programs, media interventions, policy and regulation, and tailored surveillance and evaluation. This includes surveillance of tobacco use, targeted outreach and awareness campaigns, access to culturally appropriate tobacco use dependence treatments, and efforts to restrict disproportionate marketing to sexual minority communities by the tobacco industry [22]. Gender and sexual minority inclusion, engagement, and voices should be uplifted in local, state, and national tobacco prevention and control activities.

## Data Availability

The data that support the findings of this study are available from the Public Health Survey Research Program, California State University, Sacramento but restrictions apply to the availability of these, which were used under license for the current study, and so are not publicly available. Data are however available from the authors upon reasonable request and with permission of the Public Health Survey Research Program, California State University, Sacramento.

## Abbreviations

AOR: Adjusted Odds Ratio
CA BRFSS: California Behavioral Risk Factor Surveillance System
CI: Confidence Interval
LGB: Lesbian, Gay, or Bisexual
LGBT: Lesbian, Gay, Bisexual, or Transgender
